# Metal‐based antimicrobial strategies

**DOI:** 10.1111/1751-7915.12785

**Published:** 2017-07-26

**Authors:** Raymond J. Turner

**Affiliations:** ^1^ Faculty of Science Department of Biological Sciences University of Calgary Calgary AB Canada

## Abstract

Metal based‐antimicrobials have potential for profiling sustainability solutions to infection care and health; with biotechnological applications providing novel compounds. Yet they must be used wisely for sustainable use in human and agricultural health with thoughts towards bioremediation for recovery should be considered.

The use of metal compounds as antimicrobial agents stretches back thousands of years and into the 20th century, only to be replaced by the introduction of organic antibiotics in the mid‐20th century (Hobman and Crossman, 2015). Metal‐based antimicrobials (MBA) show promise for sustainability towards communicable diseases (UN sustainable development goals ‐ 3.3), yet their use and practices influence other SDG's including 3.9, 6.3 and 12.4 that all relate to non‐polluted environments for healthy living.

The ultimate goal of antimicrobials is high efficacy at low dosage without the evolution of resistance. A renewed interest in metals as antimicrobial and biocidal agents is reflected in hopes that less resistance will evolve. Traditional antibiotics tend to follow the bullet‐target concept, acting on specific biochemical processes: replication, transcription, translation and other housekeeping metabolic enzymes, which provide ease of progressive resistance (Tenover, 2006; Aminov, 2010). Alternatively, metals appear to target multiple cellular processes leading to pleiotropic effects on bacterial cells (Lemire *et al*., 2013).

It is now common knowledge that a variety of metal ions are toxic to bacteria (Nies, 1999; Harrison *et al*., 2004). Overall, the metals that are being increasingly considered for antimicrobial agents are typically within the transition metals of the d‐block, (V, Ti, Cr, Co, Ni, Cu, Zn, Tb, W, Ag, Cd, Au, Hg) and a few other metals and metalloids from groups 13–16 of the periodic table (Al, Ga, Ge, As, Se, Sn, Sb, Te, Pb and Bi). An interesting discovery made over 10 years ago that metals have strong efficacy against microbes growing as a biofilm (Teitzel and Parsek, 2003; Harrison *et al*., 2004). This was significant as a quintessential phenotype of biofilms is their antimicrobial resistance (Stewart and Costerton, 2001). Furthermore, metals have shown some efficacy on persister cells, the dormant variants of regular cells that were impervious to antibiotics (Harrison *et al*., 2005a,b).

We have seen wide spread commercial deployment of MBAs over the past few decades, particularly Cu and Ag. Studies have documented the efficacy and performance of metal ions for a number of medical devices and products. Below follows a few examples: wound dressings containing Ag have proven to be quite effective, demonstrating a 99% reduction in cell viability (Boonkaew *et al*., 2014). Urinary catheters coated in Ag display a significant benefit to patients with urinary tract infections, when compared to traditional alloy‐coated catheters (Rupp *et al*., 2004). Combination coatings produced through the deposition of Ag and Ti have also demonstrated decreased cell viability against *Staphylococcus aureus* and *Klebsiella pneumoniae*, while displaying no cytotoxicity to epithelial and osteoblast cells (Ewald *et al*., 2006). With increasing transmission from pathogens on various surfaces, various Cu coatings have been examined for their potential in decreasing the viability of pathogenic microorganisms; reports have demonstrated a reduction in *Listeria monocytogenesnm* (Wilks *et al*., 2006)*, Escherichia coli*, including a verocytotoxigenic *E. coli* (Wilks *et al*., 2005), *Mycobacterium tuberculosis* (Mehtar *et al*. 2008), *Salmonella enterica*,* Camplylobacter jejuni* (Faúndez *et al*., 2004), vancomycin‐resistant *Enterococci* (Warnes and Keevil, 2011), methicillin‐resistant *S. aureus* (Noyce *et al*., 2006). The viability of bacteria is on the timescales of only minutes to a few hours with Cu surface exposure, when compared to other surfaces such as stainless steel, PVC, aluminium bronze and silicon bronze. Protective respiratory face masks impregnated with copper oxide exhibit enhanced anti‐influenza biocidal activity (Borkow *et al*., 2010) and Cu‐impregnated socks have been shown to improve the healing of minor wounds and cuts in diabetic patients (Borkow *et al*., 2009). It is also now common to see Cu/Ag ionizers used for controlling *Legionella* in drinking water distribution systems in hospitals to mitigate nosocomial infections (Lin *et al*., 2011). The control of many of the organisms mentioned above is key to health sustainability as many are noted on the recent WHO priority pathogens list of which new antibiotics are urgently needed (Tacconelli *et al*., 2017).

Currently, metal‐containing compounds can be purchased in‐stores and on the Web; in fact, silver has found its way into numerous consumer products, such as clothing, deodorant, toothbrushes, drinking glasses and even silver ionizer washing machines. Companies are now offering silver coating services for a range of products, from flooring to kitchen utensils, and food storage containers, to name a few. In parallel, we also see use of copper for many of these types of products. Product advertisement includes confidence in the safety of metal use as an antimicrobial, still, despite the wealth of information published on the mechanisms of metal toxicity, in many cases the precise mechanisms by which they kill microbes, and their effect on human cells for that matter still remains unclear.

Although useful in marketing, such common uses of MBAs will lead to a loss in effectiveness, similar to the misuse and overuse of antibiotics that has led to multi‐antibiotic resistance strains and their rapid loss of efficacy. Unfortunately, it may already be too late, at least for Ag and Cu, due to their widespread deployment. Additionally, there are already numerous reports around cross‐resistance between different metal‐based antimicrobials (e.g. Cu and Ag cross‐resistance reported by Torres‐Urquidy and Bright, 2012), which can lead to multimetal resistance (MMR). Certainly, microbes, particularly growing as a biofilm, have the inherent ability to develop MMR (Harrison *et al*., 2007).

Sustainability practices of the use of MBAs should also include a discussion of waste. We already see MBAs used in the agricultural industry for livestock and crops, which leads to an increased metal load in soils and precipitation/irrigation runoff. Furthermore, there appear to be co‐occurrence and coselection of antibiotic resistance genes with metal resistance genes (Li *et al*., 2017). As a complement to biotechnology approaches, the omic's technology revolution, particularly genomics and proteomics, can provide biomarkers for resistance traits once they are identified. Ultimately, this can lead to the sustainable use of metal antimicrobials through focused/personalized application approaches, especially so where MBAs are not deployed when resistance markers are present.

Bioremediation of toxic pollutants including the metals is critical for sustained health and economic welfare. However, in most countries in the world, the legislation is weak and the moderate fines are to most industries simply considered the cost of doing business. For the most part, there is little driving force for remediation and thus, we see dig and dump or fence off practices at best. Without stronger legislation and penalties, the only way to inspire industry is added incentives towards a product in the bioremediation process. This is now becoming a possibility in the realm of bioremediation of metal and metalloid contaminants.

To mitigate this metal pollutant source, remediation strategies through microbial bioremediation should be employed. Although resistance to metals can be through decreased uptake or efflux mechanisms, other mechanisms to be exploited in biotechnology for metal bioremediation are biotransformation and precipitation along with biosorption of metals. Precipitation (through biomineralization) is an exciting prospect to recover metals from metal contaminated aquatic/marine systems (Golby *et al*., 2014). For example microbial *bio‐scrubbers* of metal processing microbe communities could be employed at municipal wastewater treatment sites where millions of dollars of precious metals are released per year (Dobson and Burgess, 2007; Westerhoff *et al*., 2015).

Recently, there has been an explosion in the development of nano‐antimicrobials based on metals (Dastjerdi and Montazer, 2010). Additionally, an exciting biotechnology using microbes as green chemical factories to produce metal nanomaterials is under development; these biofactory‐produced nanomaterials have been explored for their efficacy and value as nano‐MBA. Examples include AuNP (Maliszewska *et al*., 2014), AgNP (Fayaz *et al*., 2010), SeNP (Cremonini *et al*., 2016; Piacenza *et al*., 2017) and TeNP (Srivastava *et al*., 2015). Here, one can use bacteria for the remediation of a metal pollutant to generate novel nano‐MBA materials in a sustainable fashion. Using microbes to produce the metal nanomaterials with antimicrobial properties is a realistic biotechnological promise towards sustainability, as it takes advantage of green synthetic approaches towards stable nanomaterials, superior to their chemically synthesized counterparts.

While research to date on MBAs has considerable promise, the understanding of the toxicology of these metals on humans, livestock, crops and the (microbial)‐ecosystem as a whole is lacking. Chronic exposure is frequently ignored. To obtain a sustainable practice, policies based on both acute and chronic exposure must be systematically studied in parallel with the metals antimicrobial/biocidal properties.

## Conflict of interest

None declared.
